# Safer topical treatment for inflammation using 5α-tetrahydrocorticosterone in mouse models

**DOI:** 10.1016/j.bcp.2017.01.008

**Published:** 2017-04-01

**Authors:** Annalisa Gastaldello, Dawn E.W. Livingstone, Amber J. Abernethie, Nicola Tsang, Brian R. Walker, Patrick W.F. Hadoke, Ruth Andrew

**Affiliations:** a University/British Heart Foundation Centre for Cardiovascular Science, The Queen’s Medical Research Institute, University of Edinburgh, 47 Little France Crescent, Edinburgh EH16 4TJ, United Kingdom; b Centre for Integrative Physiology, Hugh Robson Building, University of Edinburgh, 15 George Square, Edinburgh EH8 9XD, United Kingdom

**Keywords:** Glucocorticoid, 5α-Tetrahydrocorticosterone, Dermatitis, Angiogenesis, Collagen, Wound healing

## Abstract

Use of topical glucocorticoid for inflammatory skin conditions is limited by systemic and local side-effects. This investigation addressed the hypothesis that topical 5α-tetrahydrocorticosterone (5αTHB, a corticosterone metabolite) inhibits dermal inflammation without affecting processes responsible for skin thinning and impaired wound healing. The topical anti-inflammatory properties of 5αTHB were compared with those of corticosterone in C57Bl/6 male mice with irritant dermatitis induced by croton oil, whereas its effects on angiogenesis, inflammation, and collagen deposition were investigated by subcutaneous sponge implantation. 5αTHB decreased dermal swelling and total cell infiltration associated with dermatitis similarly to corticosterone after 24 h, although at a five fold higher dose, but in contrast did not have any effects after 6 h. Pre-treatment with the glucocorticoid receptor antagonist RU486 attenuated the effect of corticosterone on swelling at 24 h, but not that of 5αTHB. After 24 h 5αTHB reduced myeloperoxidase activity (representative of neutrophil infiltration) to a greater extent than corticosterone. At equipotent anti-inflammatory doses 5αTHB suppressed angiogenesis to a limited extent, unlike corticosterone which substantially decreased angiogenesis compared to vehicle. Furthermore, 5αTHB reduced only endothelial cell recruitment in sponges whereas corticosterone also inhibited smooth muscle cell recruitment and decreased transcripts of angiogenic and inflammatory genes. Strikingly, corticosterone, but not 5αTHB, reduced collagen deposition. However, both 5αTHB and corticosterone attenuated macrophage infiltration into sponges. In conclusion, 5αTHB displays the profile of a safer topical anti-inflammatory compound. With limited effects on angiogenesis and extracellular matrix, it is less likely to impair wound healing or cause skin thinning.

## Graphical abstract

**Figure:**
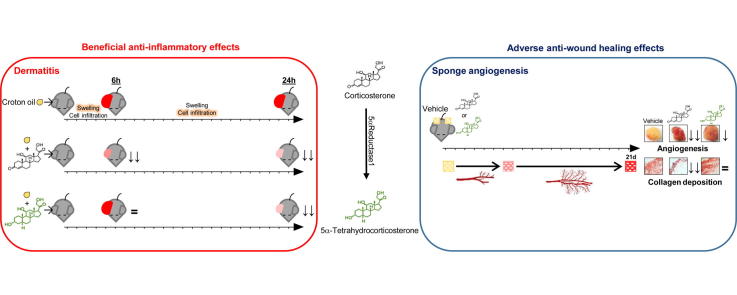

## 1 Introduction

Inflammatory skin disease is highly prevalent worldwide: according to estimates published in 2012, eczema affects approximately 230 million people globally, and topical anti-inflammatory glucocorticoids are the most common treatment [1]. However, the therapeutic benefits of these drugs are offset by severe side-effects [2].

Following application of glucocorticoids to the skin, adverse effects manifest locally (skin atrophy and impaired wound healing) and systemically (e.g. osteoporosis, abdominal obesity, and glaucoma) [3]. Skin atrophy is characterised by increased transparency and striae, due to suppression of cell proliferation and inhibition of collagen synthesis, whereas delayed wound healing is attributed to dysregulation of keratinocyte proliferation, fibroblastic activity, and angiogenesis causing delayed formation of granulation tissue [4], [5]. These factors restrict the use of more potent glucocorticoids and also the length of treatment, particularly in vulnerable patients such as infants and the elderly. Accordingly, novel drugs or delivery modes have been sought to improve the therapeutic index of topical steroids. This may be achieved by “dissociated glucocorticoids” which invoke trans-repression through the glucocorticoid receptor (GR) without trans-activation [6].

5α-Tetrahydrocorticosterone (5αTHB) is a naturally occurring glucocorticoid metabolite, formed by reduction of the A-ring of corticosterone, the principle endogenous glucocorticoid in rodents. Two 5α-reductase isozymes catalyse the rate-determining step in 5αTHB synthesis from corticosterone: the type I isozyme is expressed in the liver, kidney and skin; the type II isozyme is expressed principally in the liver, genital tract, prostate and skin. Recent studies have suggested that 5αTHB exhibits a dissociated profile of action, possibly acting through GR. 5αTHB displayed anti-inflammatory properties when administered subcutaneously in mice subjected to thioglycollate-induced peritonitis, but did not induce the chronic adverse metabolic changes (such as increased insulin and body mass) produced by corticosterone [7]. 5αTHB and corticosterone suppressed infiltration of neutrophils into the inflamed peritoneum to a similar extent, but 5αTHB had a lesser effect on macrophage recruitment, suggesting a different profile of action. When infused systemically, 5αTHB demonstrated rapid clearance from the systemic circulation [7], likely limiting its oral use, but the same characteristic would be beneficial in topical treatments in which systemic effects are unwanted.

This investigation addressed the hypothesis that topical 5αTHB can effectively reduce skin inflammation through a GR-dependent mechanism, but with limited effects on processes such as angiogenesis and collagen deposition, the inhibition of which underpin most common problems associated with topical glucocorticoid therapy. The efficacy of topical 5αTHB application for treating skin inflammation was compared with that of corticosterone *in vivo* using croton oil-induced dermatitis and the role of GR was assessed by pre-administration of the GR antagonist mifepristone (RU486). This is a steroid-responsive model of dermal inflammation, driven primarily by neutrophil accumulation, commonly used for testing anti-inflammatory treatments for irritant dermatitis [8], [9]. An *in vivo* model of sponge implantation allowed comparison of the effects of corticosterone and 5αTHB on inflammation, angiogenesis and collagen deposition.

## 2 Materials and methods

### 2.1 Materials

Chemicals were from Sigma Aldrich (Poole, UK) unless otherwise stated. Steroids (corticosterone, 5αTHB and RU486) were from Steraloids (Newport, RI, USA).

### 2.2 Animal welfare, models and ethical statements

Male mice (C57BL/6, 8–10 week old) were from Harlan Laboratories (Shardlow, UK). Animals were allowed to acclimatise for one week prior to experimentation, maintained under controlled conditions of light (lights on 0700–1900 h) and temperature (18–20 °C) and allowed free access to standard chow (Special Diet Services, Witham, UK) and drinking water. All experimental procedures were performed under UK Home Office guidelines.

#### 2.2.1 In vivo model of irritant dermatitis

Mice were treated on the inner surface of the right ear with either croton oil only (CO mixture: 10 μL, 3% v/v in ethanol and isopropyl myristate 30:5) or with solutions of corticosterone or 5αTHB diluted in the CO mixture; the left ear was untreated. The EC_50_ dose of corticosterone to reduce swelling was determined performing dose-response experiments (0.3–30 μg; 6 h, n = 4/group; 24 h, 8–12/group) in which a “non-linear log(agonist) versus response (three parameters)” regression curve was fitted using GraphPad 6 software (La Jolla, CA, USA). Subsequently, the efficacy of 5αTHB was compared using doses corresponding to 1, 3 and 5 times the EC_50_ of corticosterone after 6 h (corticosterone, 10 μg; 5αTHB, 10–50 μg; n = 4–8/group) or 24 h (corticosterone, 5 μg; 5αTHB, 5–25 μg; n = 8–12/group). Mice were culled by asphyxiation with carbon dioxide and ears excised and wet weighed. Inflammatory swelling was evaluated as the wet weight difference between treated and untreated ears, and the effect of steroids is presented as a percentage of the response to CO alone (mean weight difference = 100%).

#### 2.2.2 In vivo model of irritant dermatitis and treatment with RU486 in adrenalectomised mice

Male mice (n = 6–11/group) were injected, prior to treatment with steroids, with the GR antagonist RU486. Adrenalectomised mice were used to avoid the influence of increased physiological levels of glucocorticoids due to stress responses.

##### 2.2.2.1 Surgery

Bilateral adrenalectomy surgery was performed through dorsal incisions, under isoflurane anaesthesia (Merial, Harlow, UK). After surgery, animals were maintained on 0.9% saline drinking water to maintain fluid and electrolyte balance and allowed to recover for one week before subsequent intervention.

##### 2.2.2.2 RU486 treatments

RU486 (0.5 mg/mouse; 25 μL, 20 mg/mL in ethanol (vehicle)) was injected subcutaneously into mice 15 min prior to any topical treatment. Contact irritant dermatitis was induced and steroid treatment applied (24 h) as described in Section 2.2.1.

#### 2.2.3 In vivo model of inflammatory angiogenesis

An autoclaved cubic polyurethane sponge (∼0.5 cm^3^; grade XE1700V, Caligen Foam Ltd., Accrington, U.K.) was implanted sub-cutaneously on each flank of mice anaesthetised with isoflurane (Merial, Harlow, UK). For the delivery of steroids, one silastic pellet (Silastic 20 medical grade, Dow Corning®, Midland, USA) impregnated with vehicle (silastic only), corticosterone (3 mg/pellet) or 5αTHB (3 or 15 mg/pellet) was inserted in each sponge. Validation of the preparation and release properties of these pellets has been reported [10]. Each animal in the treatment groups had an intervention-impregnated sponge (corticosterone or 5αTHB) implanted on the right side, and a vehicle sponge on the left. Animals in the control group had vehicle sponges implanted on each side. 21 days after surgery, mice were euthanized, sponges excised and pellets removed. Sponges were bisected and one half was preserved in 10% formalin (in PBS) and the other immersed in RNAlater solution.

### 2.3 Laboratory analysis

#### 2.3.1 Histological analysis

Paraffin-embedded ear tissue and sponge slices (5 μm) were re-hydrated and stained with haematoxylin and eosin (H&E) or with Picrosirius red solution (PRS) for collagen content analysis (sponges only), prior to dehydration and mounting. Images were captured using the software QCapture Pro 7 (QImaging, Canada). On H&E stained ear sections the width of the dermis (in μm) and cell infiltration (number of nuclei) were assessed as further measurements of swelling and inflammation at both sides of the central cartilage layer. For each parameter three different areas were analysed and the mean value calculated.

#### 2.3.2 Quantitation of neutrophils by myeloperoxidase activity

Myeloperoxidase **(**MPO) activity in ear tissue was quantified using a fluorometric detection kit (ENZO Life Sciences (Exeter, UK)).

#### 2.3.3 Quantification of angiogenesis, macrophage infiltration and collagen content in sponges

For each sponge section vessels were counted in three different areas at magnification 100X. Vessels in sections stained with H&E were recognized by their round appearance and the presence of erythrocytes in the lumen. Vessels identified in sections immunostained for CD31 and αSMA marker were counted. The numbers of newly formed vessels and vessels positive for either CD31 or αSMA are represented as percentage of the vehicle group (mean value = 100%). Macrophages were counted as the number of cells positive for the marker F4/80 and expressed as a proportion of the total number of cells (blue nuclei; magnification 200X). PRS staining was quantified calculating the number of red pixels at a magnification of 100X using ImageJ software (NIH, USA).

#### 2.3.4 Immunostaining for CD31, αSMA and F4/80 in sponges

Immunostaining of sponge slices was performed using a Leica Staining Robot with robotic antigen retrieval for F4/80 using trypsin solution (0.5 mg/mL in PBS, 10 min, 37 °C), or for αSMA manual antibody retrieval using a pressure cooker under standard conditions (citrate NCL pH 6 buffer); antigen retrieval was not performed for CD31. For F4/80 and αSMA, samples were blocked (5 min) with hydrogen peroxide solution from the Leica Refine Detection Kit (Leica Biosystems, Milton Keynes, UK) followed by serum block (30 min; F4/80, Immpress anti-rat (mouse absorbed) kit (Vector Laboratories, Peterborough, UK); αSMA, Mouse on Mouse Abcam Kit (Cambridge, UK)). Thereafter, samples were incubated with primary antibody (30 min; F4/80 1:300 (eBiosciences, Hatfield, UK); αSMA 1:4000 (Sigma-Aldrich, Dorset, UK)) prior to incubation with polymer (30 min, F4/80 Impress Kit as above; αSMA as above). For CD31 a Leica Refine Kit (as above) was used, which included a hydrogen peroxide block (5 min), primary antibody (120 min, 1:200 (Abcam)) and polymer incubation (15 min). Immunostaining was completed with incubation with 3,3′-diaminobenzidine (10 min) and counterstaining with haematoxylin (5 min), both from the Leica Refine Kit.

#### 2.3.5 Total RNA extraction and PCR

Total RNA was extracted from ears and sponges using the RNeasy Fibrous Tissue Mini Kit, and first strand cDNA synthesis was performed using the QuantiTect Reverse Transcription kit (Qiagen Ltd, West Sussex, UK). Real-time quantitative PCR was performed using a LightCycler®480 (Roche Diagnostics, Mannheim, Germany) with primers (6 pmol each, Table 1), corresponding 5′ FAM-labelled probe (UPL, 2 pmol) and LightCycler®480 Probes Master [11]. Data were normalized for the mean of the transcript abundance of two housekeeping genes, TATA-binding protein and glyceraldehyde 3-phosphate dehydrogenase, the abundances of which did not differ between groups, and represented as percentage of the respective control group whose mean value was set to 100%.

### 2.4 Data analysis

All data were analysed using GraphPad Prism6 software and presented as mean ± SEM, using statistical tests as indicated. Significant differences were reported when p ⩽ 0.05. For histological analysis, the operator was blinded to treatment.

## 3 Results

### 3.1 5αTHB reduces croton oil-induced inflammation with a different time course than corticosterone

**Fig. 1:**
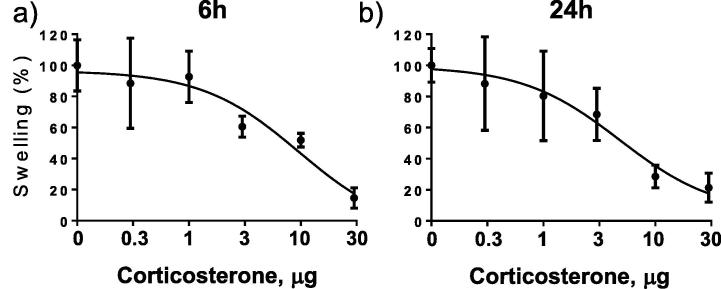
Corticosterone reduced croton oil-induced swelling in mouse ears in a dose-dependent manner at 6 and 24 h in a model of irritant dermatitis. Reduction in inflammatory swelling by increasing dose (μg) of corticosterone after (a) 6 and (b) 24 h of treatment with croton oil (6 h, n = 4/group; 24 h, n = 8–12/group). Data are mean ± SEM. Corticosterone was applied to mouse ears in a volume of 10 μL vehicle.

**Fig. 2:**
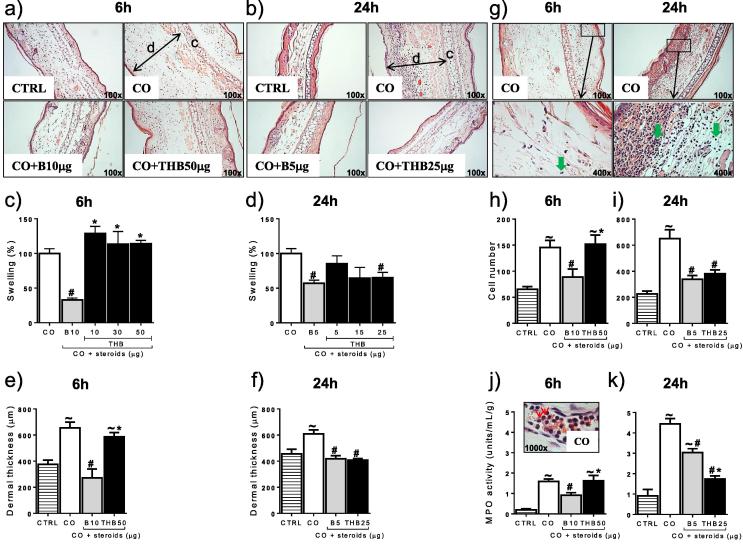
Corticosterone (B) and 5α-tetrahydrocoticosterone (THB) reduced swelling, dermal width, cell infiltration and myeloperoxidase (MPO) activity in mouse ears with a different time course in a model of croton oil (CO)-induced irritant dermatitis. Representative images of ear tissue stained with H&E showing inflammatory swelling and changes in dermal thickness (a and b) and cell infiltration (g) after treatment with CO alone or together with steroids for 6 or 24 h, as indicated; c = cartilage & d = dermis. Magnification = 100x and 400x as indicated on the images; green arrows in (g) point to cell nuclei of infiltrating cells. Quantification of swelling (c, d), dermal thickness (e, f), cell number (h, i) and MPO activity (j, k) in ears of mice after treatment for 6 or 24 h with CO alone or combined with steroids as indicated. CTRL = control ears. In (j) a representative high magnification (1000x) image of neutrophils (red arrows) in a blood vessel of a CO-treated ear is provided. The dose of each steroid in the figure refers to the total dose applied to the mouse ear in a volume of 10 μL; B 5/10 = B 5/10 μg, THB 5/10/15/25/30/50 = 5αTHB 5–50 μg; data (mean ± SEM) were analysed by one-way ANOVA followed by Tukey’s post hoc test; in (c) n = 4–8/group, in (d) n = 8–12/group; in (e), (f), (h), (i), (j) and (k) n = 6–10 tissue sections/group; p ⩽ 0.05, ∼ vs CTRL, # vs CO, ^*^ vs B.

Increasing concentrations of corticosterone reduced croton oil-induced ear swelling in a dose-dependent manner after 6 and 24 h (Fig. 1a, b), with EC_50_ doses of 10 μg at 6 h and 5 μg at 24 h. 5αTHB reduced swelling at 24 h (EC_50_ dose: 25 μg) but not at 6 h (Fig. 2a–d). Only samples treated with the highest doses of 5αTHB (50 μg at 6 h and 25 μg at 24 h) were subject to further analysis.

Histological and biochemical analyses showed that croton oil-induced swelling was accompanied by enlargement of the dermal layer at both 6 and 24 h (Fig. 2a, b, e, f), cell infiltration (Fig. 2g–i) and increased MPO activity (Fig. 2j, k). Application of corticosterone reduced dermal width at 6 and 24 h but 5αTHB only had this effect at 24 h (Fig. 2a, b, e, f). Corticosterone and 5αTHB reduced cell accumulation to a similar extent at 24 h, although corticosterone also had this effect after only 6 h (Fig. 2h, i). Moreover, 5αTHB was more effective than corticosterone in suppressing MPO activity, an indicator of neutrophil activity, after 24 h, but did not have any effect at 6 h (Fig. 2j, k).

**Fig. 3:**
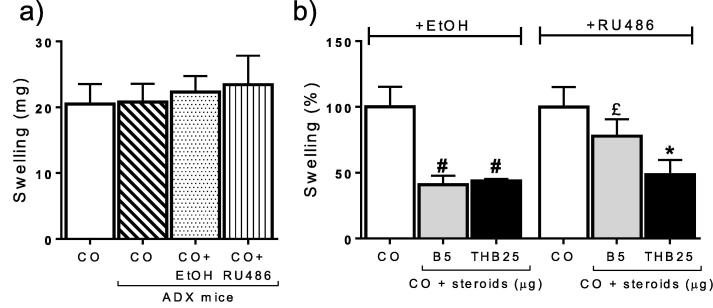
The glucocorticoid receptor antagonist RU486 attenuated the anti-inflammatory effect of corticosterone (B) but not of 5α-tetrahydrocoticosterone (THB) on croton oil (CO)-induced ear swelling at 24 h in a model of irritant dermatitis in adrenalectomised (ADX) mice. (a) Comparable swelling (mg) induced by CO in ears of non-ADX versus ADX mice treated with CO alone or in combination with ethanol (vehicle, EtOH) or RU486. (b) CO-induced ear swelling in ADX mice treated either with CO alone or together with steroids after injection of either EtOH or RU486. Numbers after steroid refer to the total dose in μg applied to the ear in a volume of 10 μL. Data (mean ± SEM) were analysed by one-way ANOVA followed by Tukey’s post hoc test; n = 7–11/group; p ⩽ 0.05, # vs CO + EtOH, ^*^ vs CO + RU486, £ vs CO + B5 + EtOH.

The involvement of GR in mediating the anti-inflammatory effects of corticosterone and 5αTHB was tested in adrenalectomised mice injected with the GR antagonist RU486. Neither adrenalectomy nor ethanol nor RU486 injection influenced the extent of CO-induced swelling (Fig. 3a). Injection of RU486 attenuated the anti-inflammatory effect of corticosterone but not that of 5αTHB (Fig. 3b).

**Fig. 4:**
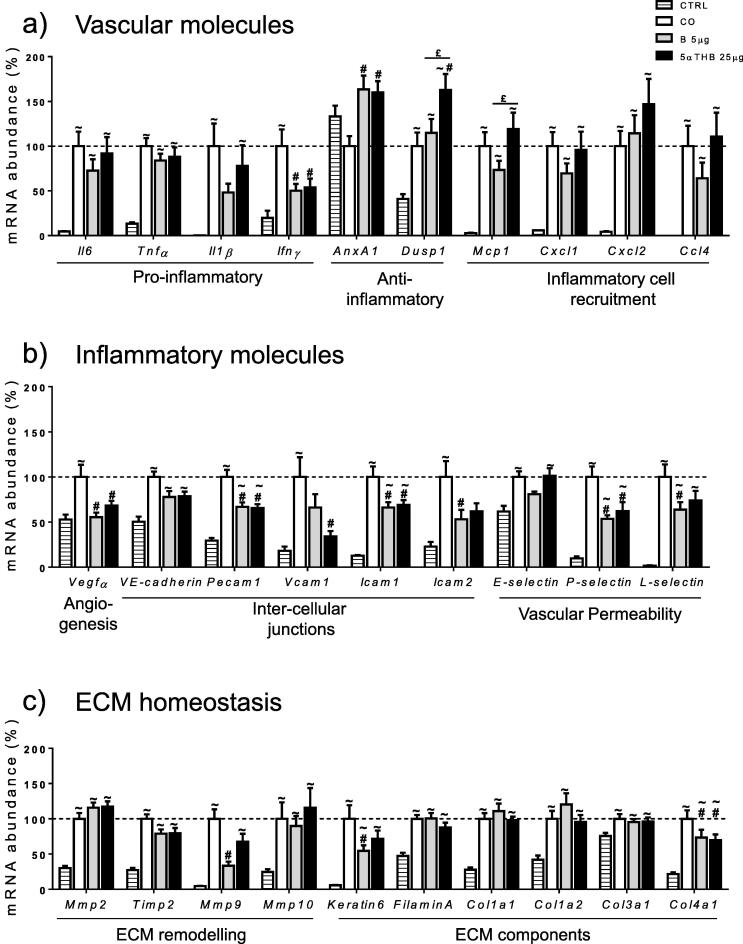
Corticosterone (B) and 5α-tetrahydrocoticosterone (5αTHB) had limited but similar effects on a variety of gene transcripts in mouse ears in a model of croton oil (CO)-induce irritant dermatitis after 24 h. Real-time PCR analysis of transcripts for (a) inflammatory, (b) vascular and (c) extra-cellular matrix (ECM) genes in control ears (CTRL) or ears treated for 24 h with either CO alone or together with B 5 μg or 5αTHB 25 μg. Data (mean ± SEM) were analysed with one-way ANOVA followed by Tukey’s post hoc test; n = 10/group; p ⩽ 0.05, # = vs CO, ∼ = vs CTRL, £ = B vs 5αTHB.

At 24 h, croton oil increased all transcripts characterising inflammatory responses, altered vascular permeability and extra-cellular matrix (ECM) regulation, with the exception of *AnxA1* (Fig. 4a–c). Corticosterone reduced abundance of *Ifnγ*, *Vegf*α, *Pecam1*, *Icam1*, *Icam2*, *P*- and *L-selectin*, *Mmp9*, *Keratin6* and *Col4a1* and increased abundance of *AnxA1* compared to the group receiving croton oil alone. 5αTHB caused similar changes to those of corticosterone, with the exception of *Icam2*, *L-selectin*, *Mmp9* and *Keratin6* which were not affected; additionally, 5αTHB increased transcripts of the anti-inflammatory gene *Dusp1* and decreased those of *Vcam1*.

#### 3.2 5αTHB and corticosterone have discrepant effects on de novo angiogenesis in sponges

**Fig. 5:**
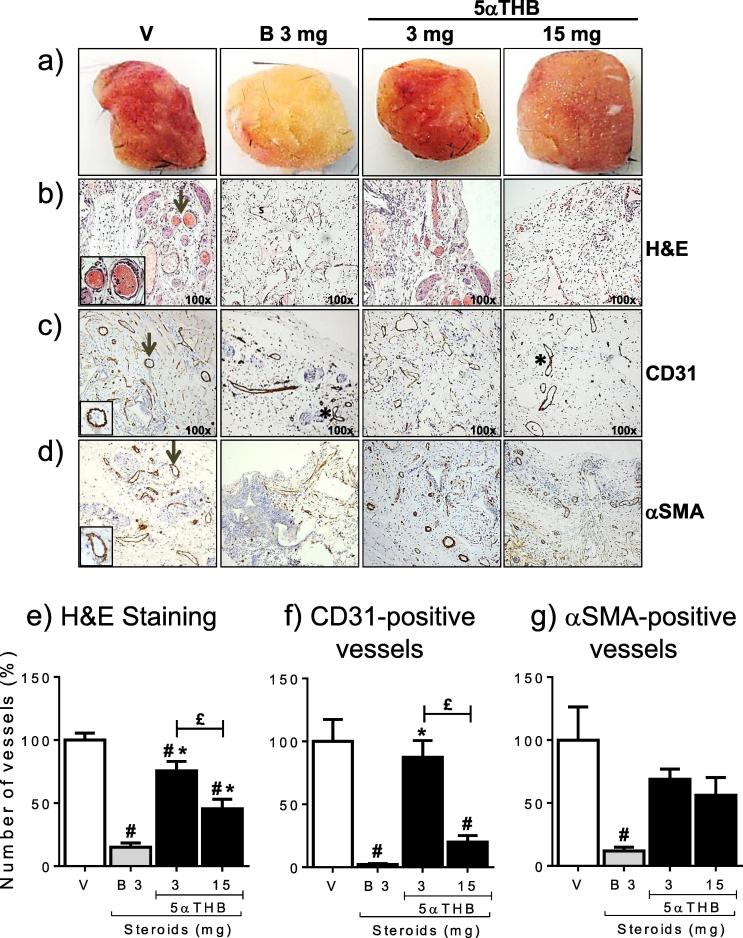
Corticosterone (B) but not 5α-tetrahydrocorticosterone (5αTHB) dramatically reduced vessel number in a model of in vivo angiogenesis. (a) Representative macroscopic pictures of sponges retrieved from mice after 20 days in situ, and containing either vehicle pellets (V) or pellets loaded with either B (3 mg) or 5αTHB (3 mg & 15 mg) as indicated. Representative microscopic images (magnification = 100x) of sections of sponges stained with (b) haematoxylin and eosin (H&E) or immunostained for (c) the endothelial marker CD31 or (d) for the smooth muscle cell marker αSMA. Black arrows point either to vessels recognisable by H&E staining or positive for the marker indicated and magnified in the corresponding inset (400x) of each image; ^*^ = examples of vessels undergoing sprouting angiogenesis. Quantification of newly formed vessels in sponges containing either vehicle (V), B or 5αTHB and stained with (e) H&E or positive for (f) CD31 or (g) αSMA. Numbers on the x axis refer to the amount (mg) of steroids in each sponge; data (mean ± SEM) were analysed with one-way ANOVA followed by Tukey’s post hoc test; n = 8–12 mice/group; p ⩽ 0.05, # = vs V, ^*^ = vs B, £ = 5αTHB 3 mg vs 15 mg.

Sponges containing vehicle pellets (V) were bright red, suggesting extensive vascularization, whereas those with corticosterone pellets (3 mg) were pale (similar to pre-implantation sponges) indicating less vascularization (Fig. 5a). Sponges with 5αTHB (3 mg) had a similar appearance to vehicle-treated sponges, whereas those with 15 mg 5αTHB were somewhat paler, although the lack of vascularization was not as extensive as that shown by sponges containing corticosterone.

**Fig. 6:**
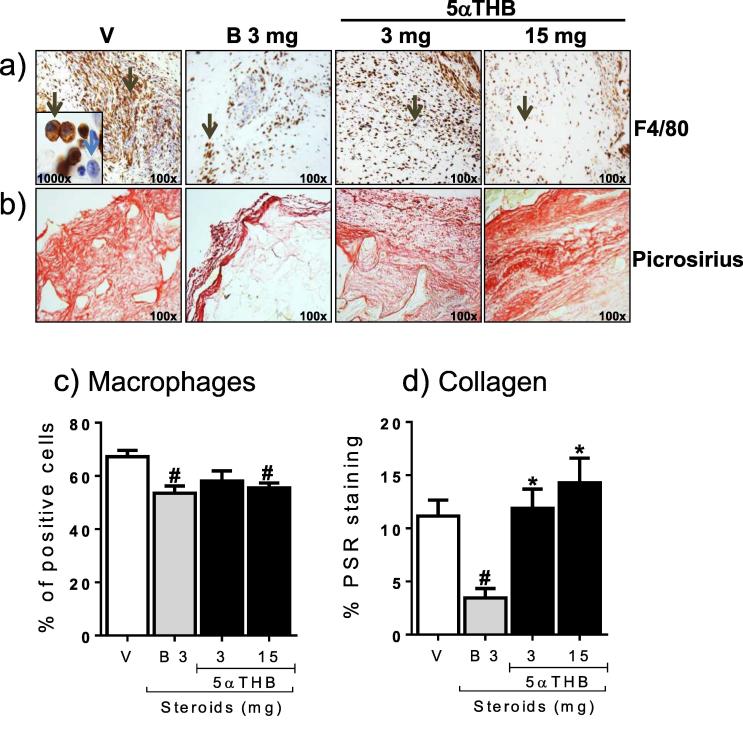
Corticosterone (B) and 5α-tetrahydrocorticosterone (5αTHB) decreased macrophage infiltration in sponges but only B reduced collagen content. Representative microscopic images (magnification 100x) of sections of sponges stained for (a) the macrophage marker F4/80 or for (b) collagen with Picrosirius red and containing either vehicle (V), B (3 mg) or 5αTHB (3 and 15 mg) as indicated. Black arrows in (a) point to cells expressing F4/80; the blue arrow in the inset (1000x) identifies the nucleus of a cell negative for F4/80. Quantification of (c) cells positive for F4/80 and (d) collagen content in sponges containing either V, B or 5αTHB; numbers on the x axis refer to the amount (mg) of steroids in each sponge. Data (mean ± SEM) were analysed with one-way ANOVA followed by Tukey’s post hoc test; n = 8–12 mice/group; p ⩽ 0.05, # = vs V, ^*^ = B vs 5αTHB.

Vessels were identified by H&E staining (Fig. 5b), and immunoreactivity for CD31 (Fig. 5c) and αSMA (Fig. 5d) associated with vascular structures. Quantification showed that corticosterone dramatically reduced vessel density whether assessed by H&E (Fig. 5e), CD31 (Fig. 5f) or αSMA (Fig. 5g) staining. In contrast, an equivalent dose (3 mg) of 5αTHB in sponges had little effect on vascular density (Fig. 5e, f, g). The higher dose (15 mg: equipotent in anti-inflammatory model) reduced vascular density assessed by H&E (Fig. 5e) and CD31 (Fig. 5f) staining but less dramatically than with corticosterone. Strikingly, 5αTHB had little effect on the density of αSMA positive vessels (Fig. 5g). Macrophage infiltration of sponges was observed (Fig. 6a, c) and was reduced following treatment with corticosterone or 5αTHB, although this did not achieve significance at the lower dose of 5αTHB (Fig. 6c). Collagen staining (Fig. 6b, d) was lower in sponges treated with corticosterone, compared with vehicle-treated controls, whereas 5αTHB had no effect at either concentration.

**Fig. 7:**
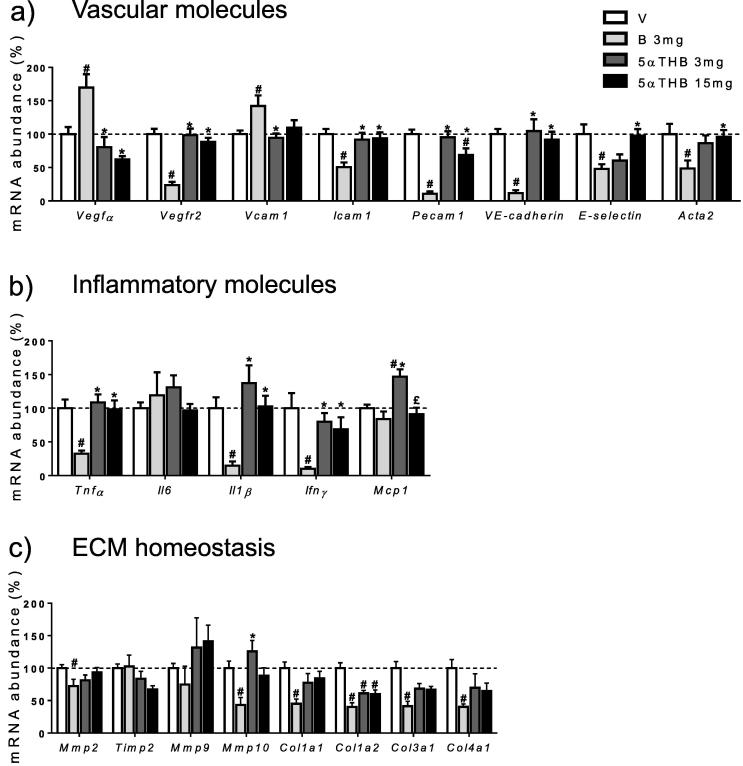
Corticosterone (B) affected gene transcripts in sponges to a greater degree than 5α-tetrahydrocorticosterone (5αTHB). Real-time PCR analysis of transcripts of (a) vascular, (b) inflammatory and (c) extra-cellular matrix (ECM) genes in sponges containing either vehicle (V), B 3 mg or 5αTHB 3 and 15 mg as indicated. Data (mean ± SEM) were analysed with one-way ANOVA followed by Tukey’s post hoc test; n = 8–12 mice/group; p ⩽ 0.05, # = vs V, ^*^ = vs B, £ = 5αTHB 3 mg vs 15 mg.

Corticosterone treatment was associated with increased transcripts for *Vegf*α and *Vcam1* whereas transcripts for *Vegfr2*, *Icam1*, *Pecam1* (CD31), *VE-cadherin*, *E-selectin* and *Acta2* (αSMA) were decreased (Fig. 7a). In contrast, 5αTHB only decreased transcripts of *Pecam1*. Corticosterone reduced the transcript abundance for the inflammatory genes *Tnf*α, *Il1β* and *Ifnγ* but had no effect on *Mcp1* or *Il6* (Fig. 7b). In contrast, neither concentration of 5αTHB reduced any of the gene transcripts, with the lower concentration (3 mg) in fact increasing the abundance of transcripts for *Mcp1* (Fig. 7b). In sponges containing corticosterone, transcripts for two matrix metalloproteinases (*Mmp2; Mmp10)* and four collagen types (*Cola1a1*, *Col1a2*, *Col3a1*, *Col4a1*) were reduced compared to vehicle (Fig. 7c) but tissue inhibitor of metalloproteinases *(Timp1*) and matrix metalloproteinase *Mmp9* was unchanged*.* In contrast, 5αTHB (3 mg & 15 mg) had limited effects, only decreasing *Col1a2*.

## 4 Discussion

Anti-inflammatory properties, but a safer systemic side effect profile, of 5αTHB, an A-ring reduced metabolite of corticosterone, have been demonstrated previously *in vivo* in a murine model of thioglycollate-induced peritonitis [7]. This investigation addressed the hypotheses that 5αTHB has advantages over conventional glucocorticoids as a topical treatment for irritant dermatitis, and that its actions are mediated through GR. 5αTHB was demonstrated to be an effective topical anti-inflammatory agent in an *in vivo* model of irritant dermatitis induced by croton oil, with effects mainly on pathways regulating vascular permeability to cell infiltration rather than suppression of pro-inflammatory markers. However, experiments using the GR antagonist RU486 suggested these effects may not be mediated by occupation of GR. Furthermore, in comparison with corticosterone, use of 5αTHB may be associated with fewer adverse local effects (less inhibition of angiogenesis and collagen deposition).

The direct suppression of inflammation by topical 5αTHB was assessed using croton oil-induced irritant dermatitis, a well-characterised, steroid-responsive model, used extensively in the pharmaceutical industry [6], [12], [13], [14]. Croton oil promotes the release of cytokines and chemokines from keratinocytes and other cells, stimulating in turn the release of pro-inflammatory cytokines such as IL1*β* and IL6 from epidermal and dermal cells, causing swelling and infiltration of leukocytes [15], [16], [17], [18]. The predominant effect at the early stages of the process is tissue oedema which peaks 6 h-post treatment; this phase is followed by cell infiltration (mainly neutrophils) which is at its highest between 12 and 24 h [19], [20], [21], [22]. This time course of changes was recapitulated here: at 6 h croton oil produced a marked swelling accompanied by cell infiltration and increased MPO activity, whereas at 24 h swelling persisted but was reduced, while cell infiltration and MPO activity increased further.

This model has been used extensively to test novel anti-inflammatory drugs, including glucocorticoids (e.g. dexamethasone, betamethasone and hydrocortisone) [21], [23], [24], [25], [26]. Here, the effects of 5αTHB on skin inflammation were compared with those of corticosterone, the active rodent glucocorticoid. Previous reports of corticosterone efficacy were not available but our data suggest that at 6 h it is twice as potent as one of the most frequently employed topical glucocorticoids, hydrocortisone [21], [27]. Notably, the dose-dependent reduction of dermal inflammation by 5αTHB exhibited a slower time course than corticosterone with no effect at 6 h. Significant suppression of inflammation was, however, achieved at 24 h, albeit requiring a 3–5 fold higher dose than corticosterone to match efficacy. This suggests that 5αTHB may predominantly target cell recruitment, which is prominent at 24 h post CO-treatment, with neutrophils believed to be the most abundant cell type present [19], [20], [21], [22]. Analysis of MPO activity showed that both steroids reduced neutrophil infiltration; interestingly, this was suppressed more effectively by 5αTHB than by corticosterone at 24 h. The lack of response to 5αTHB to reduce swelling at 6 h was accompanied by a failure to reduce cell number.

Experiments with the GR antagonist RU486 suggested that the anti-inflammatory effect of B on swelling is mediated by GR. However, this seems not to be the case for 5αTHB. The fact that the anti-inflammatory actions of 5αTHB were not antagonised by RU486 was unexpected, albeit in keeping with its different time course of action to corticosterone. 5αTHB is a glucocorticoid metabolite, previously shown capable of displacing dexamethasone from rat GR *in vitro* [28]. RU486 acts as a GR antagonist by binding to the ligand-binding domain, blocking the recruitment of essential GR co-activators [29], [30], and if 5αTHB does operate through GR it does so by an unconventional mechanism, perhaps through allosteric means. Further genetic and pharmacological studies are now required to elucidate the mechanism of action of 5αTHB.

To start to address this, the profile of action of 5αTHB was explored further through expression profiling and compared with responses produced by corticosterone. At 24 h, the impact of 5αTHB on expression of genes involved in regulation of inflammation and vascular homeostasis in the CO model was strikingly similar to those of corticosterone. A noted difference was in the upregulation of *Dusp1* by 5αTHB in contrast to corticosterone. DUSP1 is a negative regulator of the MAPK signal transduction pathway, and is increased in response to inflammatory and irritant stimuli, leading to decreased production of cytokines, chemokines and adhesion molecules [31], [32]. A glucocorticoid-mediated increase in its protein abundance has been reported previously in many cell types [33], [34], [35], [36], [37]. DUSP1 is also upregulated by non-steroidal anti-inflammatory compounds [32] and by new selective modulators of GR [38]. 5αTHB and corticosterone also increased transcripts of the anti-inflammatory gene *AnxA1* (lipocortin). Lipocortin was originally described as being responsible for the anti-inflammatory properties of glucocorticoids [39], again diminishing neutrophil recruitment by suppressing adhesion to endothelial cells [39]. Both corticosterone and 5αTHB strongly suppressed many factors involved with tissue permeability and cell recruitment. The effects of 5αTHB on expression of ECM proteins were less marked than those of corticosterone, whereas both lacked effects on transcription of most collagen genes, only decreasing *Col4a1* transcripts. Type IV collagen is one of the minor components of the skin (less than 5%) [5], mainly forming the basement membrane; as a consequence both steroids are likely to have little impact on skin thickness of croton-oil treated ears after 24 h. However accurate measures of skin thickness cannot be made in the inflamed skin *in vivo*. Previous in vitro microarray studies on cultures of primary human keratinocytes showed that dexamethasone, a glucocorticoid known to cause skin thinning, decreases transcripts of *Mmp9*, *Mmp10*, *Keratin6*, *FilaminA* and *Col4a1* after 24 h incubation [40], a profile similar to that of corticosterone in our model.

Two of the major side effects of topical anti-inflammatory glucocorticoids are skin thinning and delayed wound healing, requiring careful assessment of potential adverse effects for any proposed topical steroid therapy. Angiogenesis and collagen turnover are important factors in skin homeostasis, with angiogenesis particularly important for dermal healing [41]. Following the demonstration that cortisone inhibits angiogenesis *in vitro*, a number of “angiostatic steroids” have been identified [42]. These include 5*β*-tetrahydrocortisol, one of the A-ring reduced metabolites of cortisol (the major glucocorticoid in humans). Here the impact of 5αTHB on angiogenesis, inflammation and collagen deposition was studied in response to implantation of subcutaneous sponges [43]. This model is steroid sensitive [44] and equipotent anti-inflammatory doses of corticosterone and 5αTHB (based on results from the croton-oil model) were administered in the sponges. 5αTHB reduced angiogenesis but to a lesser degree than, and with a different pattern to, corticosterone. It was notable that both compounds reduced vessel density, predominantly by reducing the number of CD31-positive vessels. However, unlike corticosterone, 5αTHB did not reduce the number of αSMA positive vessels. CD31-staining identifies endothelial cells lining vascular structures and can be used to detect small (capillaries) and larger vessels [45]. In contrast, αSMA, staining detects pericytes and smooth muscle cells, and can be used to identify larger/maturing vessels with a functional medial layer [46]. Thus, these results suggest that, although 5αTHB does reduce new vessel formation, it does not adversely affect the number of mature vessels and, hence, may be less likely to attenuate wound healing. Caveats do, however, apply in that retention of 5αTHB and corticosterone within sponges was not directly compared in this study.

In terms of local inflammation, both corticosterone and 5αTHB inhibited macrophage infiltration. Inflammatory cells, in particular monocytes/macrophages, are central players in angiogenesis as they are a source of pro-angiogenic cytokines [47]. Indeed, it is often difficult to distinguish between direct angiostatic properties and those that are a consequence of reduced inflammation. In the croton oil model there was a bigger effect of 5αTHB compared to corticosterone on neutrophil infiltration, whereas this is not true regarding the recruitment of macrophages analysed in the angiogenesis model. This suggests that neutrophils might be the preferential cellular target for 5αTHB in an inflammatory setting. Importantly, macrophages have a pivotal role in wound repair [41], and a compound with fewer inhibitory effects on their infiltration might be more desirable.

Relatively few changes in gene transcripts for factors that regulate aspects of vascular function were consistent with 5αTHB having a limited effect on the developing vasculature, with only a modest reduction in *Pecam1* which support the immunostaining data for CD31*.* In contrast, corticosterone inhibited expression of most of the factors assessed (*Icam1*, *Pecam1*, *VE-cadherin*, *E-selectin* and *Acta2*), consistent with its potent anti-angiogenic properties [44], and again reinforce the data obtained with the immunostaining of CD31 and αSMA in sponge sections. These factors contribute to stabilisation of cell-cell contact between endothelial cells [45], and the dynamics of cell rearrangement central to vessel formation [48], [49]. The only exceptions were the increased expression of *Vegf*α and *Vcam1. Vegf*α has a key role in stimulating angiogenesis [50], [51], [52], so would be expected to be reduced by corticosterone (although it is possible that the increased expression is a consequence of insufficient angiogenesis in the sponges). However, this was accompanied by reduced *Vegfr2* transcript number, consistent with inhibition of angiogenesis seen in previous studies with other glucocorticoids [53]. VEGFR2 is the main receptor responsible for the majority of the effects of VEGF on endothelial cells [53] and is associated with proliferation of new vessels during wound healing [53].

Intriguingly, despite its potent anti-inflammatory properties, corticosterone only produced a small reduction in transcripts of pro-inflammatory genes. 5αTHB, despite reducing macrophage infiltration, did not reduce expression of any of the transcripts analysed and, indeed, increased abundance of transcripts of *Mcp1*. A similar induction of *Mcp1/MCP1* promoted vessel growth in infarcted hearts in mice [54], suggesting this may be a compensatory mechanism in response to an ischemic event. In previous studies of peritonitis 5αTHB also suppressed inflammatory cell recruitment, but with a more profound effect on neutrophils compared with macrophages [7].

The extra-cellular matrix (ECM) plays a vital role in wound healing and many studies show that glucocorticoids cause skin damage by depleting collagen isoforms [55]. This again could be modelled in the implanted sponges with consistent reductions in collagen deposition and expression of the four collagen isoform transcripts analysed in corticosterone-treated sponges. Importantly, 5αTHB had little effect on collagen staining, significantly reducing only *Col1a2* expression. Skin collagen fibrils are made mostly of type 1 collagen, a heterodimer composed by three alpha chains, two encoded by *Col1a1* and one by *Col1a2* [5]. The lack of effects on *Col1a1* transcripts by 5αTHB might explain why this compound did not affect the overall collagen content in sponges. Furthermore, the absence of effect on *Col4a1* transcripts suggest a reduced impact, compared to corticosterone, on angiogenesis, as type IV collagen is a major component of the basement membrane (facilitating the differentiation of endothelial cells during formation of capillary-like structures) [56]. This contrasts with the dermatitis model where both steroids had a small effect to reduce *Col4a1*, but over a shorter time course. Metalloproteinase enzymes (MMPs) influence wound healing, predominantly by promoting ECM degradation and cell migration [57], whereas tissue inhibitors of MMPs (TIMPs) oppose these actions. Corticosterone, but not 5αTHB, decreased expression of MMPs (*Mmp10* and *Mmp2*) within the sponges, which may contribute to alterations in matrix composition and to inhibition of angiogenesis [58], [59].

Studies of mechanism in the croton oil and sponge model were mainly limited to analysis of transcript abundance, due to paucity of tissue. While complementary analysis confirmed effects on CD31 (*Pecam1*), αSMA (*Acta2*) and collagen protein, GCs have been shown to inhibit translation rather than transcription for some molecules involved in inflammation [60], [61]. As a consequence, while no significant effect of 5αTHB or B was found on transcription of cytokines, it remains possible that they may be affecting the translation of those transcripts.

In conclusion, 5αTHB is an effective topical anti-inflammatory steroid, reducing acute skin inflammation possibly binding to a different receptor than GR. Its limited effects on angiogenesis and *de novo* deposition of collagen (combined with previous reports of its rapid systemic elimination and, therefore, reduced metabolic side-effects), suggest it may have potential as a novel topical anti-inflammatory treatment and careful dissection of its mechanism of action is now required.

## Conflicts of interest

RA and BRW are inventors on a relevant patent held by the University of Edinburgh.

## Figures and Tables

**Table 1:** Assay details for real-time PCR of murine samples. UPL denotes Universal Probe Library fluorescent probe number (Roche Diagnostics Ltd, Burgess Hill, UK). Gene names: *Acta2* = actin, alpha 2, smooth muscle; *Anxa1* = annexin A1; *Ccl4* = chemokine (C-C motif) ligand 4; *Col1a1*, *Col1a2*, *Col3a1*, *Col4a1* = collagen, type 1-3-4, alpha 1-2; *Cxcl* = chemokine (C-X-C motif) ligand; *Dusp1* = dual specificity phosphatase 1; *E-selectin* = selectin, endothelial; *Gapdh* = glyceraldehyde 3-phosphate dehydrogenase; *Icam* = intercellular adhesion molecule; *Il* = interleukin; *Ifnγ* = interferon gamma; *L-selectin* = selectin, lymphocytes; *Mcp1* = monocyte chemoattractant protein 1; *Mmp* = matrix metallopeptidase; *Pecam1* = platelet/endothelial cell adhesion molecule 1; *P-selectin* = selectin, platelets; *Tbp* = TATA-binding protein: *Timp2* = tissue inhibitor of metalloproteinase 2; *Tnf* α = tumour necrosis factor alpha; *VE-cadherin* = vascular-endothelial cadherin; *Vcam1* = vascular cell adhesion molecule 1; *Vegf*α = vascular endothelial growth factor alpha; *Vegfr2* = vascular endothelial growth factor receptor 2.

Gene Symbol	Forward primer	Reverse Primer	UPL
*Acta2*	ctctcttccagccatctttcat	tataggtggtttcgtggatgc	58
*Anxa1*	ctttgccaagccatcctg	tgggatgtctagtttccacca	21
*Ccl4*	ccagcagtctttgctccaa	gctcactggggttagcaca	34
*Col1a1*	acctaagggtaccgctgga	tccagcttctccatctttgc	19
*Col1a2*	cacctggtcctgttggaagt	caccagggaagccagtca	9
*Col3a1*	tcccctggaatctgtgaatc	tgagtcgaattggggagaat	49
*Col4a1*	agttggaggaatgggcttg	ccagggacaccctgtgag	80
*Cxcl1*	gactccagccacactccaac	tgacagcgcagctcattg	83
*Cxcl2*	ccctggttcagaaaatcatcc	cttccgttgagggacagc	63
*Dusp1*	tggttcaacgaggctattgac	ggcaatgaacaaacactctcc	89
*E-selectin*	acagcagggcaacatgaaat	caactggacccattttggaa	48
*FilaminA*	tccctcagtcctttcaggtg	gcactttgacctgcagtgg	78
*Gapdh*	aggcaaaagacaccgtcaag	agaagatgcggctgtctctg	52
*Icam1*	ttggagctagcggaccag	ccggagctgaaaagttgtaga	80
*Icam2*	gcactcggagagtctcaaca	gctgcagcttcagtgtgact	75
*Il1β*	tgtaatgaaagacggcacacc	tcttctttgggtattgcttgg	78
*Il6*	gctaccaaactggatataatcagga	ccaggtagctatggtactccagaa	6
*Ifnγ*	ctcaggaagcggaaaagga	aaaattcaaatagtgctggcaga	60
*Keratin6*	gccaaggcagacagtctaaca	caggctacggttgttgtcc	55
*L-selectin*	tggtcatctccagagccaat	gcagtccatggtacccaact	47
*Mcp1*	catccacgtgttggctca	gatcatcttgctggtgaatgagt	62
*Mmp2*	tgcagggtggtggtcatag	tcacgctcttgagactttgg	78
*Mmp9*	cagaggtaacccacgtcagc	gggatccaccttctgagactt	7
*Mmp10*	gagtctggctcatgcctacc	caggaataagttggtccctga	81
*Pecam1*	cggtgttcagcgagatcc	actcgacaggatggaaatcac	45
*P-selectin*	agcgttgcaatgtccagagt	ggatccgagcagttcacct	48
*Tbp*	gggagaatcatggaccagaa	gatgggaattccaggagtca	97
*Timp2*	aggtaccagatgggctgtga	gtccatccagaggcactcat	52
*Tnf*α	ttgagatccatgccgttg	ctgtagcccacgtcgtagc	25
*VE-cadherin*	tcaccttctgtgaggagatgg	gatgatcagcaaggtaatcactgt	6
*Vcam1*	tcttacctgtgcgctgtgac	gacctccacctgggttctct	47
*Vegf*α	aaaaacgaaagcgcaagaaa	tttctccgctctgaacaagg	1
*Vegfr2*	accagagaccctcgttttca	catttgcttgcaggaggttt	22
